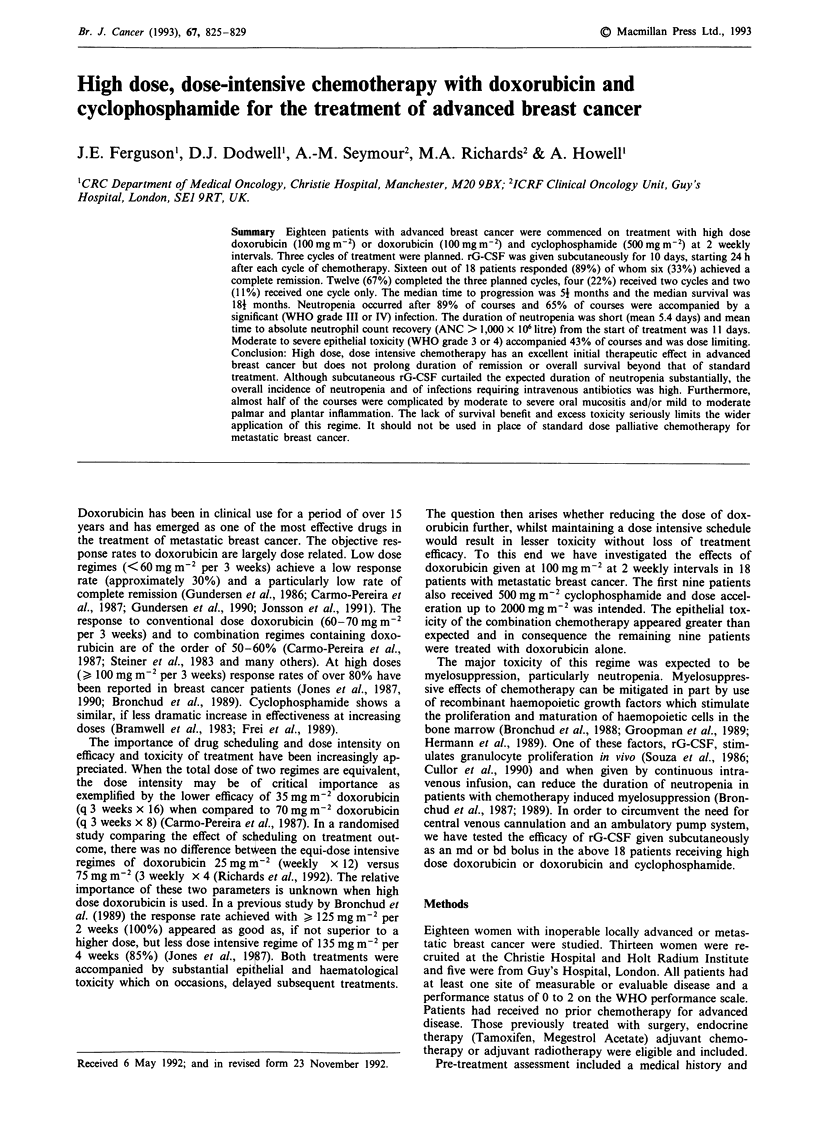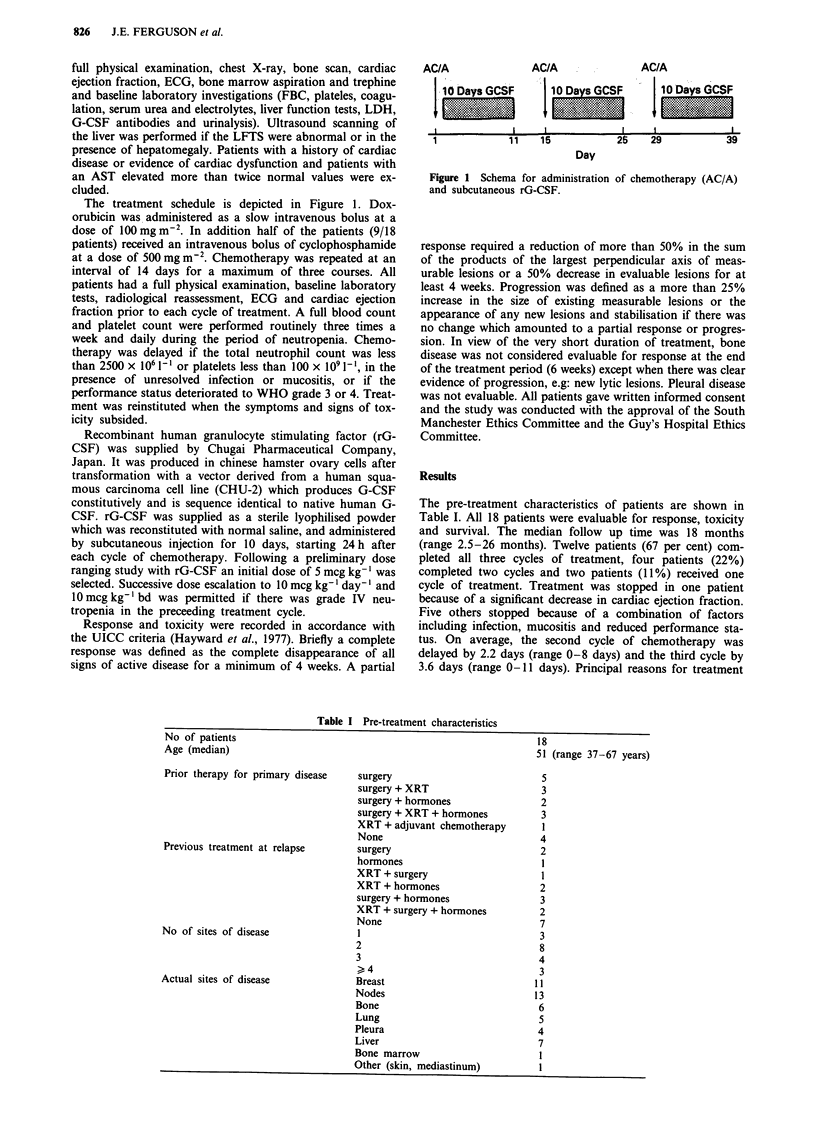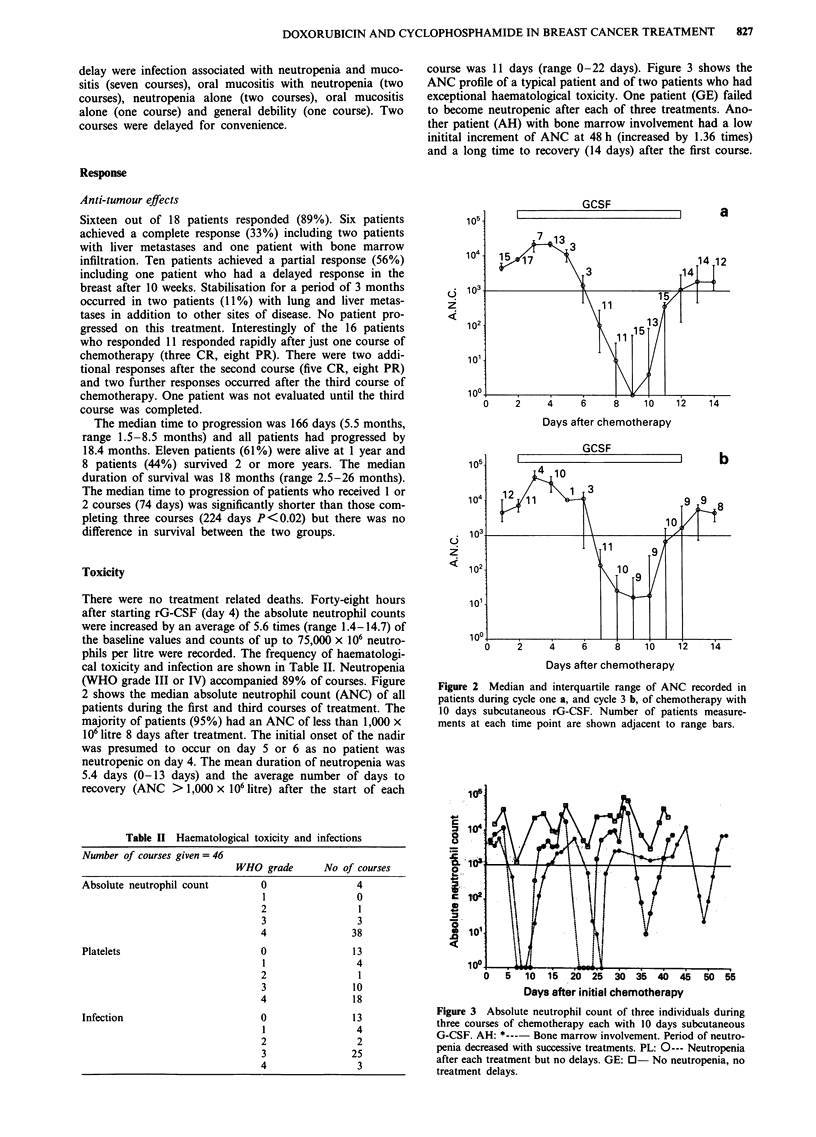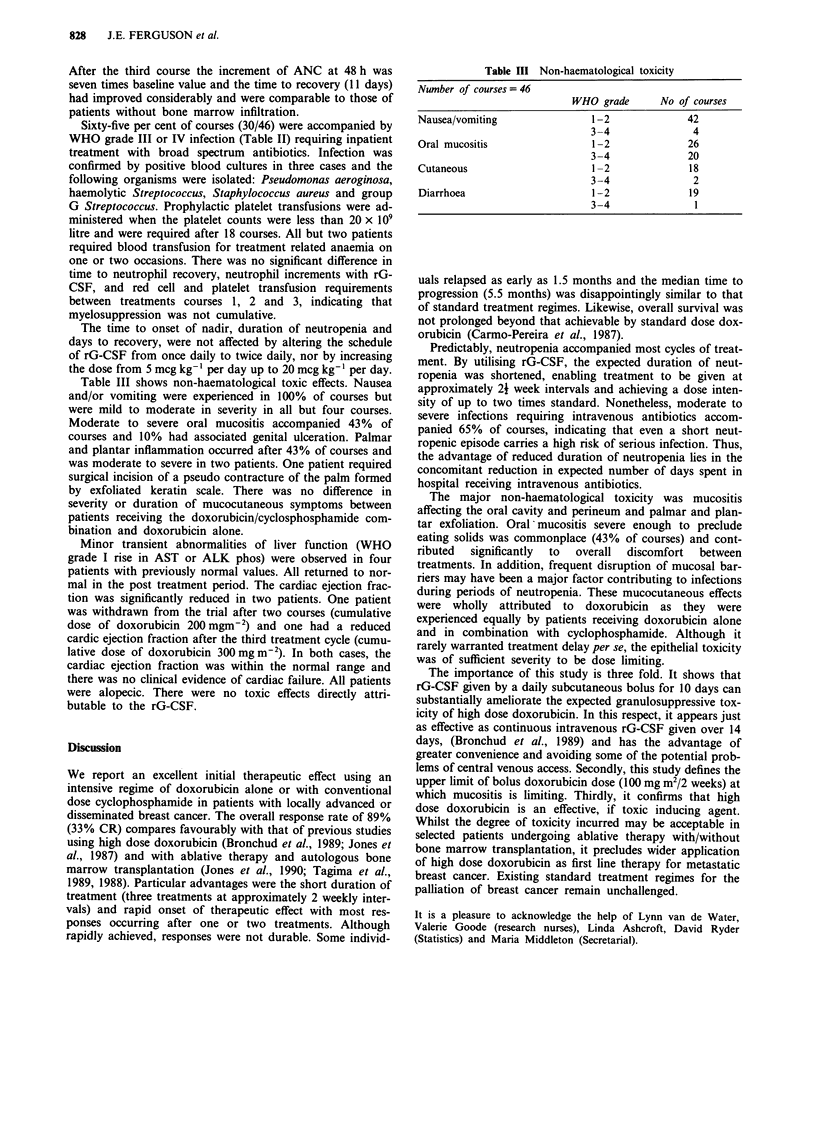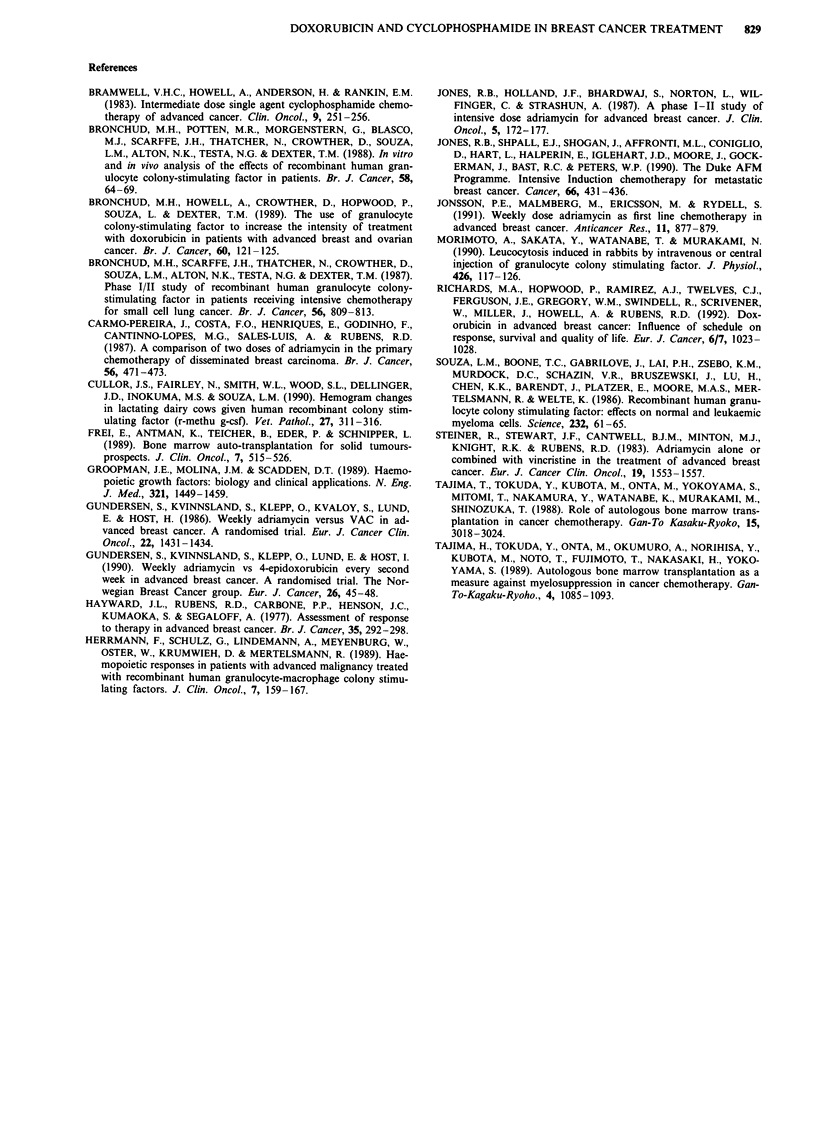# High dose, dose-intensive chemotherapy with doxorubicin and cyclophosphamide for the treatment of advanced breast cancer.

**DOI:** 10.1038/bjc.1993.151

**Published:** 1993-04

**Authors:** J. E. Ferguson, D. J. Dodwell, A. M. Seymour, M. A. Richards, A. Howell

**Affiliations:** CRC Department of Medical Oncology, Christie Hospital, Manchester, UK.

## Abstract

Eighteen patients with advanced breast cancer were commenced on treatment with high dose doxorubicin (100 mg m-2) or doxorubicin (100 mg m-2) and cyclophosphamide (500 mg m-2) at 2 weekly intervals. Three cycles of treatment were planned. rG-CSF was given subcutaneously for 10 days, starting 24 h after each cycle of chemotherapy. Sixteen out of 18 patients responded (89%) of whom six (33%) achieved a complete remission. Twelve (67%) completed the three planned cycles, four (22%) received two cycles and two (11%) received one cycle only. The median time to progression was 5 1/2 months and the median survival was 18 1/2 months. Neutropenia occurred after 89% of courses and 65% of courses were accompanied by a significant (WHO grade III or IV) infection. The duration of neutropenia was short (mean 5.4 days) and mean time to absolute neutrophil count recovery (ANC > 1,000 x 10(6) litre) from the start of treatment was 11 days. Moderate to severe epithelial toxicity (WHO grade 3 or 4) accompanied 43% of courses and was dose limiting. Conclusion: High dose, dose intensive chemotherapy has an excellent initial therapeutic effect in advanced breast cancer but does not prolong duration of remission or overall survival beyond that of standard treatment. Although subcutaneous rG-CSF curtailed the expected duration of neutropenia substantially, the overall incidence of neutropenia and of infections requiring intravenous antibiotics was high. Furthermore, almost half of the courses were complicated by moderate to severe oral mucositis and/or mild to moderate palmar and plantar inflammation. The lack of survival benefit and excess toxicity seriously limits the wider application of this regime. It should not be used in place of standard dose palliative chemotherapy for metastatic breast cancer.


Br. J. Cancer (1993), 67, 825–829 © Macmillan Press Ltd., 1993

### High dose, dose-intensive chemotherapy with doxorubicin and cyclophosphamide for the treatment of advanced breast cancer

J.E. Ferguson[1], D.J. Dodwell[1], A.-M. Seymour[2], M.A. Richards[2] & A. Howell[1]

[1]*CRC Department of Medical Oncology, Christie Hospital, Manchester, M20 9BX;* [2]*ICRF Clinical Oncology Unit, Guy's Hospital, London, SE1 9RT, UK.*

**Summary** Eighteen patients with advanced breast cancer were commenced on treatment with high dose doxorubicin (100 mg m$^{-2}$) or doxorubicin (100 mg m$^{-2}$) and cyclophosphamide (500 mg m$^{-2}$) at 2 weekly intervals. Three cycles of treatment were planned. rG-CSF was given subcutaneously for 10 days, starting 24 h after each cycle of chemotherapy. Sixteen out of 18 patients responded (89%) of whom six (33%) achieved a complete remission. Twelve (67%) completed the three planned cycles, four (22%) received two cycles and two (11%) received one cycle only. The median time to progression was 5½ months and the median survival was 18½ months. Neutropenia occurred after 89% of courses and 65% of courses were accompanied by a significant (WHO grade III or IV) infection. The duration of neutropenia was short (mean 5.4 days) and mean time to absolute neutrophil count recovery (ANC > 1,000 × 10$^6$ litre) from the start of treatment was 11 days. Moderate to severe epithelial toxicity (WHO grade 3 or 4) accompanied 43% of courses and was dose limiting. Conclusion: High dose, dose intensive chemotherapy has an excellent initial therapeutic effect in advanced breast cancer but does not prolong duration of remission or overall survival beyond that of standard treatment. Although subcutaneous rG-CSF curtailed the expected duration of neutropenia substantially, the overall incidence of neutropenia and of infections requiring intravenous antibiotics was high. Furthermore, almost half of the courses were complicated by moderate to severe oral mucositis and/or mild to moderate palmar and plantar inflammation. The lack of survival benefit and excess toxicity seriously limits the wider application of this regime. It should not be used in place of standard dose palliative chemotherapy for metastatic breast cancer.

Doxorubicin has been in clinical use for a period of over 15 years and has emerged as one of the most effective drugs in the treatment of metastatic breast cancer. The objective response rates to doxorubicin are largely dose related. Low dose regimes (< 60 mg m$^{-2}$ per 3 weeks) achieve a low response rate (approximately 30%) and a particularly low rate of complete remission (Gundersen *et al.*, 1986; Carmo-Pereira *et al.*, 1987; Gundersen *et al.*, 1990; Jonsson *et al.*, 1991). The response to conventional dose doxorubicin (60–70 mg m$^{-2}$ per 3 weeks) and to combination regimes containing doxorubicin are of the order of 50–60% (Carmo-Pereira *et al.*, 1987; Steiner *et al.*, 1983 and many others). At high doses (≥ 100 mg m$^{-2}$ per 3 weeks) response rates of over 80% have been reported in breast cancer patients (Jones *et al.*, 1987, 1990; Bronchud *et al.*, 1989). Cyclophosphamide shows a similar, if less dramatic increase in effectiveness at increasing doses (Bramwell *et al.*, 1983; Frei *et al.*, 1989).

The importance of drug scheduling and dose intensity on efficacy and toxicity of treatment have been increasingly appreciated. When the total dose of two regimes are equivalent, the dose intensity may be of critical importance as exemplified by the lower efficacy of 35 mg m$^{-2}$ doxorubicin (q 3 weeks × 16) when compared to 70 mg m$^{-2}$ doxorubicin (q 3 weeks × 8) (Carmo-Pereira *et al.*, 1987). In a randomised study comparing the effect of scheduling on treatment outcome, there was no difference between the equi-dose intensive regimes of doxorubicin 25 mg m$^{-2}$ (weekly × 12) versus 75 mg m$^{-2}$ (3 weekly × 4 (Richards *et al.*, 1992). The relative importance of these two parameters is unknown when high dose doxorubicin is used. In a previous study by Bronchud *et al.* (1989) the response rate achieved with ≥ 125 mg m$^{-2}$ per 2 weeks (100%) appeared as good as, if not superior to a higher dose, but less dose intensive regime of 135 mg m$^{-2}$ per 4 weeks (85%) (Jones *et al.*, 1987). Both treatments were accompanied by substantial epithelial and haematological toxicity which on occasions, delayed subsequent treatments.

Received 6 May 1992; and in revised form 23 November 1992.

The question then arises whether reducing the dose of doxorubicin further, whilst maintaining a dose intensive schedule would result in lesser toxicity without loss of treatment efficacy. To this end we have investigated the effects of doxorubicin given at 100 mg m$^{-2}$ at 2 weekly intervals in 18 patients with metastatic breast cancer. The first nine patients also received 500 mg m$^{-2}$ cyclophosphamide and dose acceleration up to 2000 mg m$^{-2}$ was intended. The epithelial toxicity of the combination chemotherapy appeared greater than expected and in consequence the remaining nine patients were treated with doxorubicin alone.

The major toxicity of this regime was expected to be myelosuppression, particularly neutropenia. Myelosuppressive effects of chemotherapy can be mitigated in part by use of recombinant haemopoietic growth factors which stimulate the proliferation and maturation of haemopoietic cells in the bone marrow (Bronchud *et al.*, 1988; Groopman *et al.*, 1989; Hermann *et al.*, 1989). One of these factors, rG-CSF, stimulates granulocyte proliferation *in vivo* (Souza *et al.*, 1986; Cullor *et al.*, 1990) and when given by continuous intravenous infusion, can reduce the duration of neutropenia in patients with chemotherapy induced myelosuppression (Bronchud *et al.*, 1987; 1989). In order to circumvent the need for central venous cannulation and an ambulatory pump system, we have tested the efficacy of rG-CSF given subcutaneously as an md or bd bolus in the above 18 patients receiving high dose doxorubicin or doxorubicin and cyclophosphamide.

### Methods

Eighteen women with inoperable locally advanced or metastatic breast cancer were studied. Thirteen women were recruited at the Christie Hospital and Holt Radium Institute and five were from Guy's Hospital, London. All patients had at least one site of measurable or evaluable disease and a performance status of 0 to 2 on the WHO performance scale. Patients had received no prior chemotherapy for advanced disease. Those previously treated with surgery, endocrine therapy (Tamoxifen, Megestrol Acetate) adjuvant chemotherapy or adjuvant radiotherapy were eligible and included.

Pre-treatment assessment included a medical history and full physical examination, chest X-ray, bone scan, cardiac ejection fraction, ECG, bone marrow aspiration and trephine and baseline laboratory investigations (FBC, plateles, coagulation, serum urea and electrolytes, liver function tests, LDH, G-CSF antibodies and urinalysis). Ultrasound scanning of the liver was performed if the LFTS were abnormal or in the presence of hepatomegaly. Patients with a history of cardiac disease or evidence of cardiac dysfunction and patients with an AST elevated more than twice normal values were excluded.

The treatment schedule is depicted in Figure 1. Doxorubicin was administered as a slow intravenous bolus at a dose of $100 \, mg \, m^{-2}$. In addition half of the patients (9/18 patients) received an intravenous bolus of cyclophosphamide at a dose of $500 \, mg \, m^{-2}$. Chemotherapy was repeated at an interval of 14 days for a maximum of three courses. All patients had a full physical examination, baseline laboratory tests, radiological reassessment, ECG and cardiac ejection fraction prior to each cycle of treatment. A full blood count and platelet count were performed routinely three times a week and daily during the period of neutropenia. Chemotherapy was delayed if the total neutrophil count was less than $2500 \times 10^6 \, l^{-1}$ or platelets less than $100 \times 10^9 \, l^{-1}$, in the presence of unresolved infection or mucositis, or if the performance status deteriorated to WHO grade 3 or 4. Treatment was reinstituted when the symptoms and signs of toxicity subsided.

Recombinant human granulocyte stimulating factor (rG-CSF) was supplied by Chugai Pharmaceutical Company, Japan. It was produced in chinese hamster ovary cells after transformation with a vector derived from a human squamous carcinoma cell line (CHU-2) which produces G-CSF constitutively and is sequence identical to native human G-CSF. rG-CSF was supplied as a sterile lyophilised powder which was reconstituted with normal saline, and administered by subcutaneous injection for 10 days, starting 24 h after each cycle of chemotherapy. Following a preliminary dose ranging study with rG-CSF an initial dose of $5 \, mcg \, kg^{-1}$ was selected. Successive dose escalation to $10 \, mcg \, kg^{-1} \, day^{-1}$ and $10 \, mcg \, kg^{-1}$ bd was permitted if there was grade IV neutropenia in the preceeding treatment cycle.

Response and toxicity were recorded in accordance with the UICC criteria (Hayward et al., 1977). Briefly a complete response was defined as the complete disappearance of all signs of active disease for a minimum of 4 weeks. A partial response required a reduction of more than 50% in the sum of the products of the largest perpendicular axis of measurable lesions or a 50% decrease in evaluable lesions for at least 4 weeks. Progression was defined as a more than 25% increase in the size of existing measurable lesions or the appearance of any new lesions and stabilisation if there was no change which amounted to a partial response or progression. In view of the very short duration of treatment, bone disease was not considered evaluable for response at the end of the treatment period (6 weeks) except when there was clear evidence of progression, e.g: new lytic lesions. Pleural disease was not evaluable. All patients gave written informed consent and the study was conducted with the approval of the South Manchester Ethics Committee and the Guy's Hospital Ethics Committee.

**Figure 1** Schema for administration of chemotherapy (AC/A) and subcutaneous rG-CSF.

### Results

The pre-treatment characteristics of patients are shown in Table I. All 18 patients were evaluable for response, toxicity and survival. The median follow up time was 18 months (range 2.5–26 months). Twelve patients (67 per cent) completed all three cycles of treatment, four patients (22%) completed two cycles and two patients (11%) received one cycle of treatment. Treatment was stopped in one patient because of a significant decrease in cardiac ejection fraction. Five others stopped because of a combination of factors including infection, mucositis and reduced performance status. On average, the second cycle of chemotherapy was delayed by 2.2 days (range 0–8 days) and the third cycle by 3.6 days (range 0–11 days). Principal reasons for treatment

**Table I** Pre-treatment characteristics

| | | |
|---|---|---|
| No of patients | | 18 |
| Age (median) | | 51 (range 37–67 years) |
| Prior therapy for primary disease | surgery | 5 |
| | surgery + XRT | 3 |
| | surgery + hormones | 2 |
| | surgery + XRT + hormones | 3 |
| | XRT + adjuvant chemotherapy | 1 |
| | None | 4 |
| Previous treatment at relapse | surgery | 2 |
| | hormones | 1 |
| | XRT + surgery | 1 |
| | XRT + hormones | 2 |
| | surgery + hormones | 3 |
| | XRT + surgery + hormones | 2 |
| | None | 7 |
| No of sites of disease | 1 | 3 |
| | 2 | 8 |
| | 3 | 4 |
| | ⩾4 | 3 |
| Actual sites of disease | Breast | 11 |
| | Nodes | 13 |
| | Bone | 6 |
| | Lung | 5 |
| | Pleura | 4 |
| | Liver | 7 |
| | Bone marrow | 1 |
| | Other (skin, mediastinum) | 1 |

delay were infection associated with neutropenia and mucositis (seven courses), oral mucositis with neutropenia (two courses), neutropenia alone (two courses), oral mucositis alone (one course) and general debility (one course). Two courses were delayed for convenience.

### Response

### *Anti-tumour effects*

Sixteen out of 18 patients responded (89%). Six patients achieved a complete response (33%) including two patients with liver metastases and one patient with bone marrow infiltration. Ten patients achieved a partial response (56%) including one patient who had a delayed response in the breast after 10 weeks. Stabilisation for a period of 3 months occurred in two patients (11%) with lung and liver metastases in addition to other sites of disease. No patient progressed on this treatment. Interestingly of the 16 patients who responded 11 responded rapidly after just one course of chemotherapy (three CR, eight PR). There were two additional responses after the second course (five CR, eight PR) and two further responses occurred after the third course of chemotherapy. One patient was not evaluated until the third course was completed.

The median time to progression was 166 days (5.5 months, range 1.5–8.5 months) and all patients had progressed by 18.4 months. Eleven patients (61%) were alive at 1 year and 8 patients (44%) survived 2 or more years. The median duration of survival was 18 months (range 2.5–26 months). The median time to progression of patients who received 1 or 2 courses (74 days) was significantly shorter than those completing three courses (224 days $P<0.02$) but there was no difference in survival between the two groups.

### Toxicity

There were no treatment related deaths. Forty-eight hours after starting rG-CSF (day 4) the absolute neutrophil counts were increased by an average of 5.6 times (range 1.4–14.7) of the baseline values and counts of up to $75,000 \times 10^6$ neutrophils per litre were recorded. The frequency of haematological toxicity and infection are shown in Table II. Neutropenia (WHO grade III or IV) accompanied 89% of courses. Figure 2 shows the median absolute neutrophil count (ANC) of all patients during the first and third courses of treatment. The majority of patients (95%) had an ANC of less than $1,000 \times 10^6$ litre 8 days after treatment. The initial onset of the nadir was presumed to occur on day 5 or 6 as no patient was neutropenic on day 4. The mean duration of neutropenia was 5.4 days (0–13 days) and the average number of days to recovery (ANC $>1,000 \times 10^6$ litre) after the start of each course was 11 days (range 0–22 days). Figure 3 shows the ANC profile of a typical patient and of two patients who had exceptional haematological toxicity. One patient (GE) failed to become neutropenic after each of three treatments. Another patient (AH) with bone marrow involvement had a low initial increment of ANC at 48 h (increased by 1.36 times) and a long time to recovery (14 days) after the first course.

**Table II** Haematological toxicity and infections

| *Number of courses given = 46* | *WHO grade* | *No of courses* |
|---|---|---|
| Absolute neutrophil count | 0 | 4 |
| | 1 | 0 |
| | 2 | 1 |
| | 3 | 3 |
| | 4 | 38 |
| Platelets | 0 | 13 |
| | 1 | 4 |
| | 2 | 1 |
| | 3 | 10 |
| | 4 | 18 |
| Infection | 0 | 13 |
| | 1 | 4 |
| | 2 | 2 |
| | 3 | 25 |
| | 4 | 3 |

**Figure 2** Median and interquartile range of ANC recorded in patients during cycle one **a**, and cycle 3 **b**, of chemotherapy with 10 days subcutaneous rG-CSF. Number of patients measurements at each time point are shown adjacent to range bars.

**Figure 3** Absolute neutrophil count of three individuals during three courses of chemotherapy each with 10 days subcutaneous G-CSF. AH: *---— Bone marrow involvement. Period of neutropenia decreased with successive treatments. PL: O--- Neutropenia after each treatment but no delays. GE: □— No neutropenia, no treatment delays.

After the third course the increment of ANC at 48 h was seven times baseline value and the time to recovery (11 days) had improved considerably and were comparable to those of patients without bone marrow infiltration.

Sixty-five per cent of courses (30/46) were accompanied by WHO grade III or IV infection (Table II) requiring inpatient treatment with broad spectrum antibiotics. Infection was confirmed by positive blood cultures in three cases and the following organisms were isolated: *Pseudomonas aeroginosa*, haemolytic *Streptococcus*, *Staphylococcus aureus* and group G *Streptococcus*. Prophylactic platelet transfusions were administered when the platelet counts were less than $20 \times 10^9$ litre and were required after 18 courses. All but two patients required blood transfusion for treatment related anaemia on one or two occasions. There was no significant difference in time to neutrophil recovery, neutrophil increments with rG-CSF, and red cell and platelet transfusion requirements between treatments courses 1, 2 and 3, indicating that myelosuppression was not cumulative.

The time to onset of nadir, duration of neutropenia and days to recovery, were not affected by altering the schedule of rG-CSF from once daily to twice daily, nor by increasing the dose from 5 mcg $kg^{-1}$ per day up to 20 mcg $kg^{-1}$ per day.

Table III shows non-haematological toxic effects. Nausea and/or vomiting were experienced in 100% of courses but were mild to moderate in severity in all but four courses. Moderate to severe oral mucositis accompanied 43% of courses and 10% had associated genital ulceration. Palmar and plantar inflammation occurred after 43% of courses and was moderate to severe in two patients. One patient required surgical incision of a pseudo contracture of the palm formed by exfoliated keratin scale. There was no difference in severity or duration of mucocutaneous symptoms between patients receiving the doxorubicin/cyclosphosphamide combination and doxorubicin alone.

Minor transient abnormalities of liver function (WHO grade I rise in AST or ALK phos) were observed in four patients with previously normal values. All returned to normal in the post treatment period. The cardiac ejection fraction was significantly reduced in two patients. One patient was withdrawn from the trial after two courses (cumulative dose of doxorubicin 200 $mgm^{-2}$) and one had a reduced cardic ejection fraction after the third treatment cycle (cumulative dose of doxorubicin 300 mg $m^{-2}$). In both cases, the cardiac ejection fraction was within the normal range and there was no clinical evidence of cardiac failure. All patients were alopecic. There were no toxic effects directly attributable to the rG-CSF.

**Table III** Non-haematological toxicity

| *Number of courses = 46* | *WHO grade* | *No of courses* |
|---|---|---|
| Nausea/vomiting | 1–2 | 42 |
| | 3–4 | 4 |
| Oral mucositis | 1–2 | 26 |
| | 3–4 | 20 |
| Cutaneous | 1–2 | 18 |
| | 3–4 | 2 |
| Diarrhoea | 1–2 | 19 |
| | 3–4 | 1 |

### Discussion

We report an excellent initial therapeutic effect using an intensive regime of doxorubicin alone or with conventional dose cyclophosphamide in patients with locally advanced or disseminated breast cancer. The overall response rate of 89% (33% CR) compares favourably with that of previous studies using high dose doxorubicin (Bronchud *et al.*, 1989; Jones *et al.*, 1987) and with ablative therapy and autologous bone marrow transplantation (Jones *et al.*, 1990; Tagima *et al.*, 1989, 1988). Particular advantages were the short duration of treatment (three treatments at approximately 2 weekly intervals) and rapid onset of therapeutic effect with most responses occurring after one or two treatments. Although rapidly achieved, responses were not durable. Some individuals relapsed as early as 1.5 months and the median time to progression (5.5 months) was disappointingly similar to that of standard treatment regimes. Likewise, overall survival was not prolonged beyond that achievable by standard dose doxorubicin (Carmo-Pereira *et al.*, 1987).

Predictably, neutropenia accompanied most cycles of treatment. By utilising rG-CSF, the expected duration of neutropenia was shortened, enabling treatment to be given at approximately 2½ week intervals and achieving a dose intensity of up to two times standard. Nonetheless, moderate to severe infections requiring intravenous antibiotics accompanied 65% of courses, indicating that even a short neutropenic episode carries a high risk of serious infection. Thus, the advantage of reduced duration of neutropenia lies in the concomitant reduction in expected number of days spent in hospital receiving intravenous antibiotics.

The major non-haematological toxicity was mucositis affecting the oral cavity and perineum and palmar and plantar exfoliation. Oral mucositis severe enough to preclude eating solids was commonplace (43% of courses) and contributed significantly to overall discomfort between treatments. In addition, frequent disruption of mucosal barriers may have been a major factor contributing to infections during periods of neutropenia. These mucocutaneous effects were wholly attributed to doxorubicin as they were experienced equally by patients receiving doxorubicin alone and in combination with cyclophosphamide. Although it rarely warranted treatment delay *per se*, the epithelial toxicity was of sufficient severity to be dose limiting.

The importance of this study is three fold. It shows that rG-CSF given by a daily subcutaneous bolus for 10 days can substantially ameliorate the expected granulosuppressive toxicity of high dose doxorubicin. In this respect, it appears just as effective as continuous intravenous rG-CSF given over 14 days, (Bronchud *et al.*, 1989) and has the advantage of greater convenience and avoiding some of the potential problems of central venous access. Secondly, this study defines the upper limit of bolus doxorubicin dose (100 mg $m^2$/2 weeks) at which mucositis is limiting. Thirdly, it confirms that high dose doxorubicin is an effective, if toxic inducing agent. Whilst the degree of toxicity incurred may be acceptable in selected patients undergoing ablative therapy with/without bone marrow transplantation, it precludes wider application of high dose doxorubicin as first line therapy for metastatic breast cancer. Existing standard treatment regimes for the palliation of breast cancer remain unchallenged.

It is a pleasure to acknowledge the help of Lynn van de Water, Valerie Goode (research nurses), Linda Ashcroft, David Ryder (Statistics) and Maria Middleton (Secretarial).

**References**

BRAMWELL, V.H.C., HOWELL, A., ANDERSON, H. & RANKIN, E.M. (1983). Intermediate dose single agent cyclophosphamide chemotherapy of advanced cancer. *Clin. Oncol.*, **9**, 251–256.

BRONCHUD, M.H., POTTEN, M.R., MORGENSTERN, G., BLASCO, M.J., SCARFFE, J.H., THATCHER, N., CROWTHER, D., SOUZA, L.M., ALTON, N.K., TESTA, N.G. & DEXTER, T.M. (1988). *In vitro* and *in vivo* analysis of the effects of recombinant human granulocyte colony-stimulating factor in patients. *Br. J. Cancer*, **58**, 64–69.

BRONCHUD, M.H., HOWELL, A., CROWTHER, D., HOPWOOD, P., SOUZA, L. & DEXTER, T.M. (1989). The use of granulocyte colony-stimulating factor to increase the intensity of treatment with doxorubicin in patients with advanced breast and ovarian cancer. *Br. J. Cancer*, **60**, 121–125.

BRONCHUD, M.H., SCARFFE, J.H., THATCHER, N., CROWTHER, D., SOUZA, L.M., ALTON, N.K., TESTA, N.G. & DEXTER, T.M. (1987). Phase I/II study of recombinant human granulocyte colony-stimulating factor in patients receiving intensive chemotherapy for small cell lung cancer. *Br. J. Cancer*, **56**, 809–813.

CARMO-PEREIRA, J., COSTA, F.O., HENRIQUES, E., GODINHO, F., CANTINNO-LOPES, M.G., SALES-LUIS, A. & RUBENS, R.D. (1987). A comparison of two doses of adriamycin in the primary chemotherapy of disseminated breast carcinoma. *Br. J. Cancer*, **56**, 471–473.

CULLOR, J.S., FAIRLEY, N., SMITH, W.L., WOOD, S.L., DELLINGER, J.D., INOKUMA, M.S. & SOUZA, L.M. (1990). Hemogram changes in lactating dairy cows given human recombinant colony stimulating factor (r-methu g-csf). *Vet. Pathol.*, **27**, 311–316.

FREI, E., ANTMAN, K., TEICHER, B., EDER, P. & SCHNIPPER, L. (1989). Bone marrow auto-transplantation for solid tumours-prospects. *J. Clin. Oncol.*, **7**, 515–526.

GROOPMAN, J.E., MOLINA, J.M. & SCADDEN, D.T. (1989). Haemopoietic growth factors: biology and clinical applications. *N. Eng. J. Med.*, **321**, 1449–1459.

GUNDERSEN, S., KVINNSLAND, S., KLEPP, O., KVALOY, S., LUND, E. & HOST, H. (1986). Weekly adriamycin versus VAC in advanced breast cancer. A randomised trial. *Eur. J. Cancer Clin. Oncol.*, **22**, 1431–1434.

GUNDERSEN, S., KVINNSLAND, S., KLEPP, O., LUND, E. & HOST, I. (1990). Weekly adriamycin vs 4-epidoxorubicin every second week in advanced breast cancer. A randomised trial. The Norwegian Breast Cancer group. *Eur. J. Cancer*, **26**, 45–48.

HAYWARD, J.L., RUBENS, R.D., CARBONE, P.P., HENSON, J.C., KUMAOKA, S. & SEGALOFF, A. (1977). Assessment of response to therapy in advanced breast cancer. *Br. J. Cancer*, **35**, 292–298.

HERRMANN, F., SCHULZ, G., LINDEMANN, A., MEYENBURG, W., OSTER, W., KRUMWIEH, D. & MERTELSMANN, R. (1989). Haemopoietic responses in patients with advanced malignancy treated with recombinant human granulocyte-macrophage colony stimulating factors. *J. Clin. Oncol.*, **7**, 159–167.

JONES, R.B., HOLLAND, J.F., BHARDWAJ, S., NORTON, L., WILFINGER, C. & STRASHUN, A. (1987). A phase I–II study of intensive dose adriamycin for advanced breast cancer. *J. Clin. Oncol.*, **5**, 172–177.

JONES, R.B., SHPALL, E.J., SHOGAN, J., AFFRONTI, M.L., CONIGLIO, D., HART, L., HALPERIN, E., IGLEHART, J.D., MOORE, J., GOCKERMAN, J., BAST, R.C. & PETERS, W.P. (1990). The Duke AFM Programme. Intensive Induction chemotherapy for metastatic breast cancer. *Cancer*, **66**, 431–436.

JONSSON, P.E., MALMBERG, M., ERICSSON, M. & RYDELL, S. (1991). Weekly dose adriamycin as first line chemotherapy in advanced breast cancer. *Anticancer Res.*, **11**, 877–879.

MORIMOTO, A., SAKATA, Y., WATANABE, T. & MURAKAMI, N. (1990). Leucocytosis induced in rabbits by intravenous or central injection of granulocyte colony stimulating factor. *J. Physiol.*, **426**, 117–126.

RICHARDS, M.A., HOPWOOD, P., RAMIREZ, A.J., TWELVES, C.J., FERGUSON, J.E., GREGORY, W.M., SWINDELL, R., SCRIVENER, W., MILLER, J., HOWELL, A. & RUBENS, R.D. (1992). Doxorubicin in advanced breast cancer: Influence of schedule on response, survival and quality of life. *Eur. J. Cancer*, **6/7**, 1023–1028.

SOUZA, L.M., BOONE, T.C., GABRILOVE, J., LAI, P.H., ZSEBO, K.M., MURDOCK, D.C., SCHAZIN, V.R., BRUSZEWSKI, J., LU, H., CHEN, K.K., BARENDT, J., PLATZER, E., MOORE, M.A.S., MERTELSMANN, R. & WELTE, K. (1986). Recombinant human granulocyte colony stimulating factor: effects on normal and leukaemic myeloma cells. *Science*, **232**, 61–65.

STEINER, R., STEWART, J.F., CANTWELL, B.J.M., MINTON, M.J., KNIGHT, R.K. & RUBENS, R.D. (1983). Adriamycin alone or combined with vincristine in the treatment of advanced breast cancer. *Eur. J. Cancer Clin. Oncol.*, **19**, 1553–1557.

TAJIMA, T., TOKUDA, Y., KUBOTA, M., ONTA, M., YOKOYAMA, S., MITOMI, T., NAKAMURA, Y., WATANABE, K., MURAKAMI, M., SHINOZUKA, T. (1988). Role of autologous bone marrow transplantation in cancer chemotherapy. *Gan-To Kasaku-Ryoko*, **15**, 3018–3024.

TAJIMA, H., TOKUDA, Y., ONTA, M., OKUMURO, A., NORIHISA, Y., KUBOTA, M., NOTO, T., FUJIMOTO, T., NAKASAKI, H., YOKOYAMA, S. (1989). Autologous bone marrow transplantation as a measure against myelosuppression in cancer chemotherapy. *Gan-To-Kagaku-Ryoho.*, **4**, 1085–1093.